# Large-Scale Gene Relocations following an Ancient Genome Triplication Associated with the Diversification of Core Eudicots

**DOI:** 10.1371/journal.pone.0155637

**Published:** 2016-05-19

**Authors:** Yupeng Wang, Stephen P. Ficklin, Xiyin Wang, F. Alex Feltus, Andrew H. Paterson

**Affiliations:** 1 Plant Genome Mapping Laboratory, University of Georgia, Athens, Georgia, United States of America; 2 Department of Horticulture, Washington State University, Pullman, Washington, United States of America; 3 Department of Genetics and Biochemistry, Clemson University, Clemson, South Carolina, United States of America; University of Guelph, CANADA

## Abstract

Different modes of gene duplication including whole-genome duplication (WGD), and tandem, proximal and dispersed duplications are widespread in angiosperm genomes. Small-scale, stochastic gene relocations and transposed gene duplications are widely accepted to be the primary mechanisms for the creation of dispersed duplicates. However, here we show that most surviving ancient dispersed duplicates in core eudicots originated from large-scale gene relocations within a narrow window of time following a genome triplication (γ) event that occurred in the stem lineage of core eudicots. We name these surviving ancient dispersed duplicates as relocated γ duplicates. In *Arabidopsis thaliana*, relocated γ, WGD and single-gene duplicates have distinct features with regard to gene functions, essentiality, and protein interactions. Relative to γ duplicates, relocated γ duplicates have higher non-synonymous substitution rates, but comparable levels of expression and regulation divergence. Thus, relocated γ duplicates should be distinguished from WGD and single-gene duplicates for evolutionary investigations. Our results suggest large-scale gene relocations following the γ event were associated with the diversification of core eudicots.

## Introduction

Gene duplication by different mechanisms is a primary raw material for the origin and evolution of new genes, as well as generating functional novelty and specialization [1]. The angiosperms (flowering plants) are an outstanding model in which to investigate the modes and consequences of gene duplication. Large-scale gene duplications such as whole-genome duplications (WGDs) have been recurring in angiosperm evolution [2–5]. *Arabidopsis*, a model angiosperm, experienced two WGDs since its divergence from other members of the Brassicales clade (αand β), and a more ancient triplication (γ) shared with all core eudicots [3, 6, 7]. Single-gene duplications in angiosperms are also widespread [8–10].

Single-gene duplications have been subdivided into local and dispersed duplications [8]. Local duplications may occur by tandem duplication (consecutive in the genome and presumed to arise through unequal crossing over) [8] and proximal duplication (near one another but separated by a few genes, thought to occur by localized transposon activities) [11–13]. Dispersed duplicates are neither adjacent to each other in the genome nor within homologous chromosomal segments [11, 12, 14]. Transposon-induced single-gene transpositions or transposed gene duplications, via either DNA or RNA-based mechanisms, have been suggested to account for the widespread existence of dispersed duplicates [8, 15–19].

Following WGDs, genomes have preferentially retained genes encoding transcription factors [20, 21]. Moreover, gene fates are often correlated across multiple WGD events [22]. For example, in *Arabidopsis*, γ duplicates are more often retained in duplicate for both β and α events [22]. In contrast to WGDs, genomes have preferentially retained local duplications of genes encoding membrane proteins or functioning in stress response [23]. In *Populus trichocarpa*, whole-genome and local duplicates were found to differ in gene functions, protein lengths and expression patterns [24]. In addition, the age distribution of the duplicates derived from a large-scale event exhibits a peak in ancient times due to dramatic increase in the number of duplicated genes, while single-gene duplicates show an *L* shaped age distribution due to a high and constant rate of gene loss [25, 26].

However, WGD and local duplicates comprise only part of the whole set of duplicated genes. A substantial part of the duplicated gene population is dispersed duplicates [8, 9]. To date, only a few things are known regarding the preservation and evolution of dispersed duplicates. Recent dispersed duplicates, often referred to as transposed duplicates, are often associated with flanking repeats, and certain classes of genes that tend to form local duplications are more likely to have transposed than other gene classes [27]. Certain *Arabidopsis* gene families that are prone to mobility have transposed in different epochs throughout the rosids [10].

WGD events have long been thought to play a major role in plant speciation [28, 29]. Recently, bioinformatics analysis of 41 plant genomes suggested that polyploidy extensively occurred around the Cretaceous–Paleogene (K–Pg) extinction event about 66 million years ago (Mya) [30]. However, following the γ genome triplication around 125 Mya, core eudicots diverged in a narrow window of time during which few WGD events occurred [6, 7, 31]. Thus, WGD itself may not explain the rapid diversification of core eudicots. In this work, we study the evolutionary origins of dispersed duplicates and explore relationships between dispersed duplicates and the diversity of core eudicots.

## Results

### A substantial proportion of the survivalγduplicates were relocated in core eudicots

**Fig 1:**
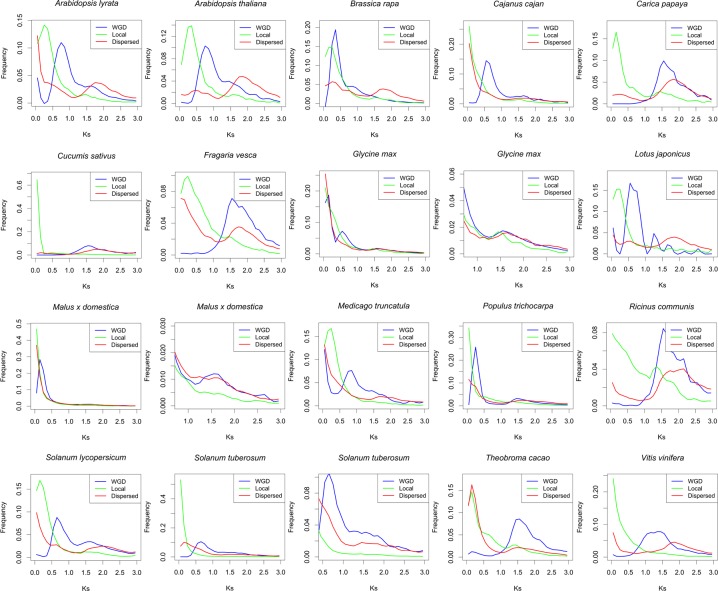
Comparison of Ks distributions among WGD, local and dispersed duplicates in the investigated core eudicot genomes. For *Glycine max*, *Solanum tuberosum* and *Malus x domestica* whose genomes are large and recent duplicates are rampant, a second plot is provided to show the right tails of Ks distributions.

We identified WGD, local and dispersed duplicates (see Methods) in 17 sequenced core eudicot genomes, including *Ricinus communis*, *Populus trichocarpa*, *Lotus japonicus*, *Medicago truncatula*, *Glycine max*, *Cajanus cajan*, *Cucumis sativus*, *Malus x domestica*, *Fragaria vesca*, *Arabidopsis thaliana*, *Arabidopsis lyrata*, *Brassica rapa*, *Carica papaya*, *Theobroma cacao*, *Vitis vinifera*, *Solanum tuberosum* and *Solanum lycopersicum*. Synonymous substitution rates (Ks) were initially used to estimate the relative ages of gene duplications. Large-scale gene duplication events such as WGDs often result in Ks peaks because of the short periods during which large numbers of duplicated genes were created [25]. In contrast, single-gene duplications such as local and transposed duplications typically show Ks distributions of *L* shape, because gene duplications and subsequent deletions are random and at relatively constant rates during evolution [26]. We compared Ks distributions among WGD, local and dispersed duplicates in these 17 core eudicot genomes (Fig 1). In all these genomes, the Ks distributions of local duplicates show an *L* shape, and the Ks distributions of WGD duplicates exhibit peaks depending on the epochs of WGD events. However, in most investigated core eudicot genomes, dispersed duplicates show a secondary Ks peak (in the few others whose genome are big and recent duplications are rampant they show a thicker Ks distribution than local duplicates) at relatively large Ks levels (1.5<Ks<2.2). Dispersed duplicates, if all of them were created by single-gene events, should show an L-shaped Ks distribution. The secondary Ks peaks/thickness at high Ks levels for dispersed duplicates in the investigated core eudicot genomes suggest that the ages of dispersed duplicates are a mixture of two distributions–“*L* shaped + a secondary peak”. We hypothesized that in a typical core eudicot genome, part of the dispersed duplicates originated from single-gene duplication events that lead to the *L* shaped distribution, while there was also a burst of dispersed duplicates created by ancient large-scale gene duplication events. Since the levels of the secondary Ks peaks are very similar among the 17 investigated core eudicot genomes, the burst of dispersed duplicates was likely to begin in the common ancestor of core eudicots. It is known that a genome triplication event (γ) occurred in the stem lineage of core eudicots [7]. It is highly possible that most of the ancient dispersed duplicates in these investigated core eudicot genomes originated from the γ event.

**Fig 2:**
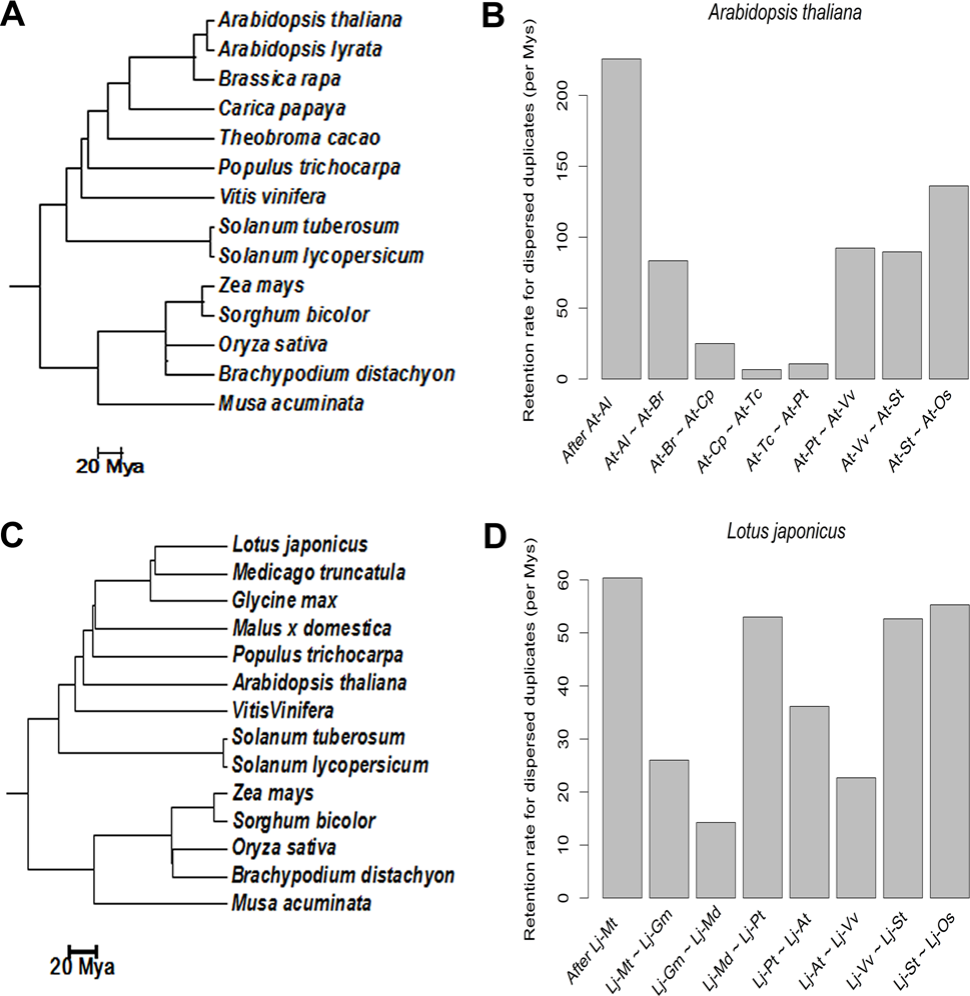
Different retention rates of dispersed duplicates during the evolution of core eudicots. (A) Phylogenetic relationships between *Arabidopsis thaliana* and its outgroups used for estimating the epochs (ages) of dispersed duplicates according to colinearity conservation. (B) Retention rates of *Arabidopsis thaliana* dispersed duplicates in different epochs. Abbreviations: At: *Arabidopsis thaliana*; Al: *Arabidopsis lyrata*; Br: *Brassica rapa*; Cp: *Carica papaya*; Tc: *Theobroma cacao*; Pt: *Populus trichocarpa*; Vv: *Vitis vinifera*; St: *Solanum tuberosum*; Os: *Oryza sativa*. Dispersed duplicates created after At-Br divergence were named transposed duplicates, while those created between At-Pt and At-St divergence were named relocated γ duplicates. (C) Phylogenetic relationships between *Lotus japonicus* and its outgroups used for estimating the epochs (ages) of dispersed duplicates according to colinearity conservation. (D) Retention rates of *Lotus japonicus* dispersed duplicates in different epochs. Abbreviations: Lj: *Lotus japonicas*; Mt: *Medicago truncatula*; Gm: *Glycine max*; Md: *Malus x domestica*; Pt: *Populus trichocarpa*; At: *Arabidopsis thaliana*; Vv: *Vitis vinifera*; St: *Solanum tuberosum*; Os: *Oryza sativa*.

To examine whether there was a burst of dispersed duplication in the early evolution of core eudicots, we investigated the detailed origins of duplicated genes in *Arabidopsis thaliana*, since the genome of *Arabidopsis thaliana* has been extensively studied. Using the procedure described in Methods, we classified *Arabidopsis thaliana* duplicated genes into 5156α, 2142β, 802γ, 3602 tandem, 1197 proximal, and 9345 dispersed duplicates. We used gene colinearity conservation between *Arabidopsis thaliana* and outgroups (phylogeny shown in Fig 2A) to estimate the epochs (ages) of *Arabidopsis thaliana* dispersed duplicates [32, 33], i.e. to examine between which speciation events each dispersed duplicate was created. For example, if an *Arabidopsis thaliana* duplicated gene shows colinearity conservation with *Populus trichocarpa*, but not with *Vitis vinifera* or more distant outgroups, this duplicated gene is estimated to be created during the epoch between *Arabidopsis-Populus* and *Arabidopsis-Vitis* divergence. Using such logic, the ages of *Arabidopsis thaliana* dispersed duplicates were assigned to eight epochs, including after *Arabidopsis thaliana*-*Arabidopsis lyrata* divergence (<5 Mya), between *Arabidopsis thaliana-Arabidopsis lyrata* and *Arabidopsis-Brassica* divergence (5~16 Mya), between *Arabidopsis-Brassica* and *Arabidopsis-Carica* divergence (16~72 Mya), between *Arabidopsis-Carica* and *Arabidopsis-Theobroma* divergence (72~90 Mya), between *Arabidopsis-Theobroma* and *Arabidopsis-Populus* divergence (90~107 Mya), between *Arabidopsis-Populus* and *Arabidopsis-Vitis* divergence (107~113 Mya), between *Arabidopsis-Vitis* and *Arabidopsis-Solanum* divergence (113~125 Mya), and between *Arabidopsis-Solanum* and *Arabidopsis-Oryza* divergence (125~148 Mya). For each epoch, we computed a retention rate for dispersed duplicates, which was the number of retained dispersed duplicates divided by the epoch length (in millions of years). We compared retention rates of dispersed duplicates across different epochs (Fig 2B), and found that there was indeed a burst of dispersed duplicates between *Arabidopsis-Populus* and *Arabidopsis-Oryza* divergence (107~148 Mya), i.e., in the early evolutionary period of core eudicots. To test whether this observation is unique to *Arabidopsis thaliana*, we also made plots for *Lotus japonicus*, which also showed a high retention rate for dispersed duplicates between *Lotus-Vitis* and *Lotus-Oryza* divergence (113~148 Mya, Fig 2C and 2D). Further, we observed very similar patterns of retention for the dispersed duplicates in *Arabidopsis lyrata* and *Medicago truncatula* (S1 and S2 Figs), based on the above phylogenies (Fig 2A and 2C). These observations support the hypothesis that dispersed gene duplications in the initial tens of millions of years following the γ event were much more frequent than before and thereafter in core eudicots. Since single-gene duplications tend to have steady deletion rates, the most plausible explanation of this burst of gene dispersal is that extensive chromosome rearrangements following the γ event relocated most γ duplicates to new positions.

We named the 4763 *Arabidopsis thaliana* dispersed duplicates generated between *Arabidopsis-Populus* and *Arabidopsis-Solanum* divergence (two epochs) as ‘relocated γ duplicates’, and reserve “γ duplicates” to denote genes remaining at the colinear positions within γ colinear blocks. Because the γ event occurred prior to *Arabidopsis-Solanum* divergence, among the dispersed duplicates generated between *Arabidopsis-Solanum* and *Arabidopsis-Oryza* divergence there might be a possibility that some were created prior to the γ event. Thus, we excluded the dispersed duplicates generated during this epoch from relocated γ duplicates. The evolutionary origins of all duplicated genes in *Arabidopsis thaliana* are listed in S1 Table. Relocated γ duplicates tend to have smaller Ks than γ duplicates (*P* = 0.047, *t*-test), precluding the possibility that most of the relocated γ duplicates were created by the ancestral duplication events of seed plants and angiosperms [5]. Additionally, we named the 2047 *Arabidopsis thaliana* dispersed duplicates created after *Arabidopsis-Brassica* divergence as “transposed duplicates”. The number of γ duplicates in *Arabidopsis thaliana* is only 802. Thus, a large proportion (85.6%) of the duplicates created by the γ event appear to have been relocated in *Arabidopsis*. It might be possible that recent WGD events have erased most signals of the γ event in *Arabidopsis*. In the lineages of *Carica Papaya* and *Theobroma cacao* whose genomes have not experienced recent WGD events, we estimated 80.2% and 47.8% of the γ duplicates were relocated respectively (see Methods). Thus, it is reasonable to conclude that a substantial proportion of the retained γ duplicates were relocated in core eudicots.

### Relocated γ duplicates are functionally distinct from WGD and single-gene duplicates in *Arabidopsis thaliana*

**Fig 3:**
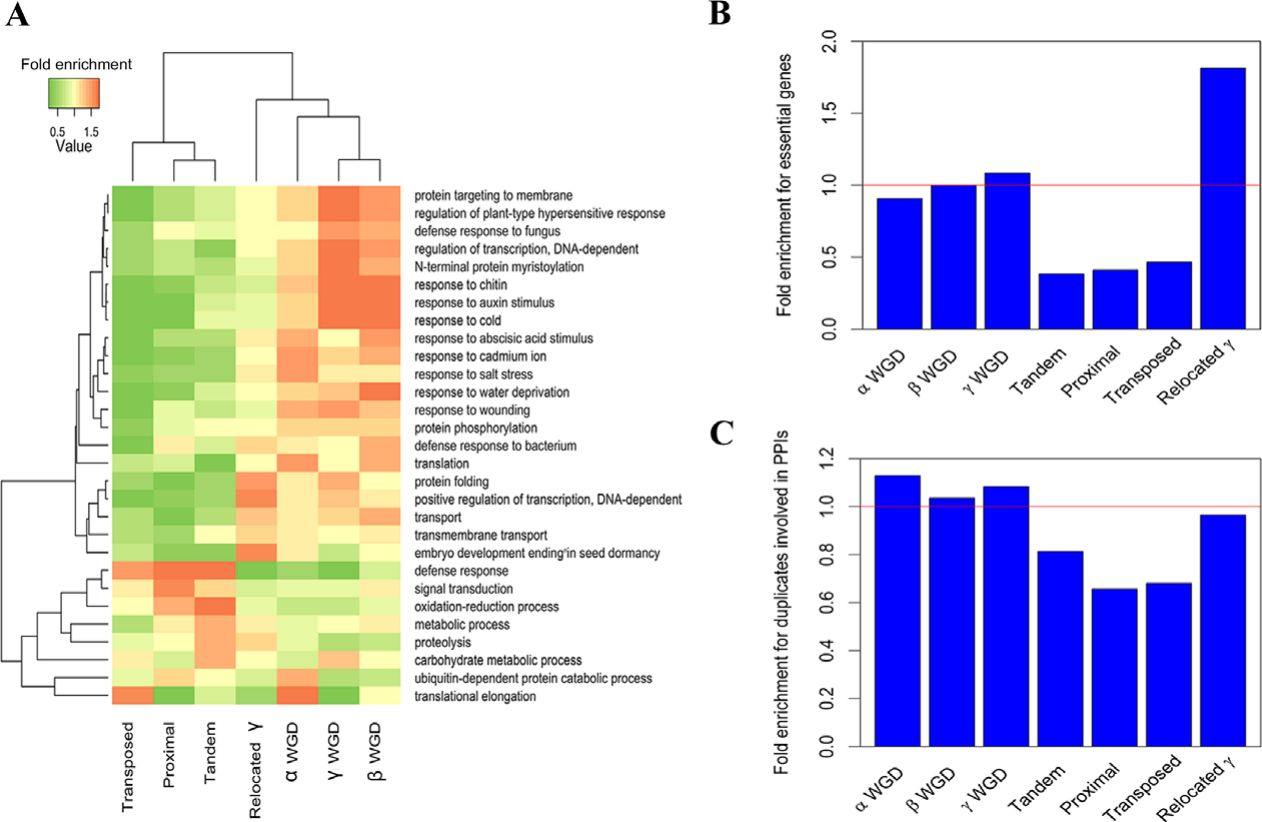
Functional comparison of different origins of duplicates. (A) Clustering of functional profiles. (B) Comparison of fold enrichment for essential genes. (C) Comparison of fold enrichment for the genes involved in protein-protein interactions (PPIs).

We investigated the functional features of relocated γ duplicates in *Arabidopsis thaliana* by comparison withα, β, γ, tandem, proximal, transposed and relocated γ duplicates. We used the Gene Ontology (GO) terms to represent the biological functions of *Arabidopsis thaliana* genes. As described in Methods, the functional profile of each origin of duplicates was denoted by its fold enrichment for the 29 biggest “biological process” GO terms (see details in S2 Table). We clustered the functional profiles of different origins of duplicated genes using a heat map (Fig 3A). Relocated γ duplicates were clustered with WGD duplicates at the second level of the dendrogram, while single-gene duplicates formed another cluster at the second level. Thus, relocated γ duplicates slightly resemble WGD duplicates, but are more different from single-gene duplicates in terms of biological functions.

Essential genes cause loss-of-function mutant phenotypes in single-gene knock-out experiments. We analyzed 2400 genes with a loss-of-function mutant phenotype in *Arabidopsis thaliana* [34]. Fold enrichment of essential genes in different origins of duplicates was compared (Fig 3B). Relocated γ duplicates are significantly enriched for essential genes (*P*<2.2×10^−16^), α duplicates are only slightly enriched for essential genes (*P* = 0.04), β and γ duplicates are not distinguishable from random probabilities of being essential genes (*P*>0.1), while single-gene duplicates are depleted for essential genes (*P*<3.0×10^−9^). This observation suggests that relocated γ duplicates tend to perform important biological functions, which are not likely to be compensated by other genes.

The gene balance hypothesis suggests that any surviving genome has retained an optimal balance of gene products that bind with one another to form protein complexes, or are involved in multiple steps of biological pathways [35]. In terms of network connectivity, more connected gene products should be more essential as phenotype is more likely to change if dosage imbalance happens [8]. According to this hypothesis, WGD duplicates should be more frequently involved in protein-protein interactions (PPIs) than single-gene duplications. Using a large-scale PPI data [36], we compared the enrichment of duplicates involved in PPIs among different origins of duplicates (Fig 3C), which shows that α duplicates are more likely to be involved in PPIs (*P* = 0.64×10^−4^); single-gene duplicates including tandem, proximal and transposed duplicates are less likely to be involved in PPIs (*P*<0.005); and β, γ and relocated γ duplicates have close-to-average probabilities of involvement in PPIs (*P*>0.1). In partial summary, the comparisons of biological functions, essentiality and PPIs among different origins of duplicated genes in *Arabidopsis thaliana* suggest that in terms of functional landscape, relocated γ duplicates are distinct from WGD and single-gene duplicates.

### Evolutionary significance of gene relocations following the γ event in *Arabidopsis*

**Fig 4:**
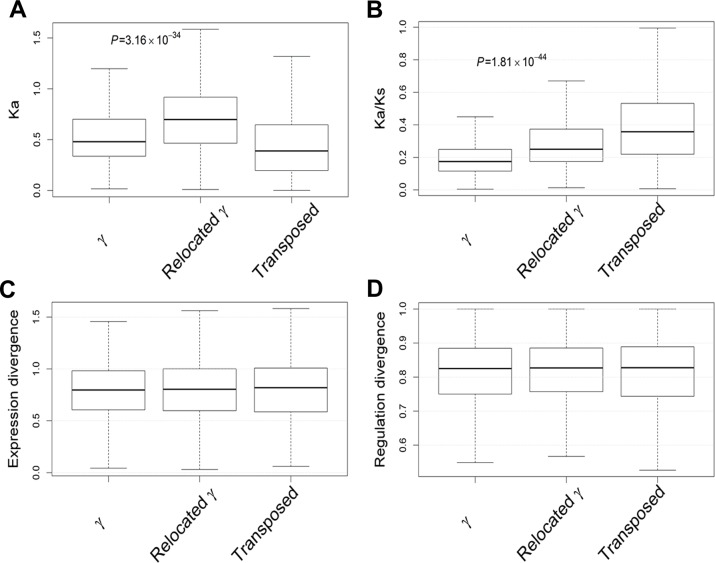
Evolutionary significance of relocated γ duplicates. (A) Comparison of Ks among γ, relocated γ and transposed duplicates. (B) Comparison of Ka/Ks among γ, relocated γ and transposed duplicates. (C) Comparison of expression divergence among γ, relocated γ and transposed duplicates. (D) Comparison of gene regulation divergence among γ, relocated γ and transposed duplicates.

We investigated the evolutionary significance of gene relocations following the γ event in *Arabidopsis thaliana*. We first examined if gene relocations following the γ event increased coding sequence divergence. We used non-synonymous substitution rates (Ka) and Ka/Ks to indicate coding sequence divergence and selection pressure respectively. Comparison of Ka among γ, relocated γ and transposed duplicates shows that relocated γ duplicates tend to code more divergent proteins than γ duplicates (Fig 4A). Comparison of Ka/Ks shows that relocated γ duplicates are under more relaxed purifying selection than γ duplicates (Fig 4B), suggesting that relocated γ duplicates are less affected by the gene balance constraints which often impose strong purifying selection on duplicated genes within tens of millions of years following WGDs [8]. However, higher Ka/Ks values for transposed duplicates may be explained by the theory that most transposed duplicates are accumulating degenerative mutations and will be ultimately pseudogenized, although admittedly there is a small chance of transposed duplicates being preserved for long times due to relaxed selective constraints and neofunctionalization [33]. Further, we investigated whether gene relocations following the γ event increased gene expression and regulation divergence. Expression divergence between γ, relocated γ and transposed duplicates is plotted in Fig 4C, showing that expression divergence is in general comparable among these three categories of duplicates (*P*>0.1, *t*-test). Furthermore, gene regulation divergence (denoted by the difference of transcription factor binding sites in their promoter regions) between γ, relocated γ and transposed duplicates is plotted in Fig 4D, showing that gene regulation divergence is also comparable among the three categories of duplicates (*P*>0.1, *t*-test). These observations suggest that the primary evolutionary impact of gene relocations following the γ event was to make duplicated genes less affected by purifying selection, perhaps rendering them freer to acquire amino acid changes and to evolve novel and critical biological functions.

## Discussion

The γ event was suggested to have occurred at the base of the core eudicots. Following the γ event, core eudicot lineages rapidly radiated, giving rise to nearly 75% of angiosperm species [6]. The large-scale gene relocations following the γ event were thus associated with the diversification of core eudicots. We observed Ks peaks around 2.0 for the dispersed duplicates in the investigated core eudicots. Due to extensive lineage-specific genome and gene duplications which are meanwhile mixed with extensive gene losses, the original pairs of ancient dispersed duplicates are largely lost in the extant genomes. However, future studies can investigate pairs of ancient dispersed duplicates which have been preserved in all core eudicot genomes and their contributions to the survival of core eudicots.

Large-scale, extensive gene relocations could provide a huge number of recombinations of genetic materials for the genomes to rapidly evolve. The number of relocated γ duplicates (4763) is almost 6 times the number of γ duplicates (802) in *Arabidopsis thaliana*. In contrast, the number of transposed duplicates (2047) is significantly smaller than that of α duplicates (5156). Thus, in *Arabidopsis thaliana*, most of the duplicates originating from the γ event were relocated shortly, while a small proportion of the duplicates originating from the α event have been relocated. In *Carica* and *Theobroma*, >47% of the γ duplicates were found to have relocated. The relocated γ duplicates we identified in *Arabidopsis thaliana* all show colinearity with *Populus trichocarpa* and/or *Vitis vinifera*, suggesting that they did not relocate again within the recent 100 million years. Thus, large-scale, extensive gene relocations may have been a singular feature of the γ event. In addition to fractionation, small-scale and mostly deleterious gene relocations are common across different WGD events [8]. A possible distinct mechanism for the gene relocations following the γ event could be that the triplicated chromosomes were fragile and underwent many rearrangements, which were then gradually fused into bigger chromosomes [37].

## Methods

### Genome annotations

Gene locations, coding sequences and colinear gene pairs for eudicot genomes including *Ricinus communis*, *Populus trichocarpa*, *Lotus japonicus*, *Medicago truncatula*, *Glycine max*, *Cajanus cajan*, *Cucumis sativus*, *Malus x domestica*, *Fragaria vesca*, *Arabidopsis lyrata*, *Arabidopsis thaliana*, *Brassica rapa*, *Carica papaya*, *Theobroma cacao*, *Vitis vinifera*, *Solanum tuberosum*, *Solanum lycopersicum*, and outgroup monocot genomes including *Oryza sativa*, *Brachypodium distachyon*, *Sorghum bicolor*, *Zea mays* and *Musa acuminata* were obtained from the Plant Genome Duplication Database (http://chibba.agtec.uga.edu/duplication/) [38]. Divergence time between species was retrieved from Time Tree [39].

### Ka and Ks computation

To generate a coding sequence alignment for a pair of duplicated genes, their protein sequences were first aligned using Clustalw [40] with default parameters. Then, the protein alignment was converted to coding sequence alignment using the “Bio::Align::Utilities” module of the BioPerl package (http://www.bioperl.org/). Ka and Ks were calculated using the Yang & Nielsen method [41] via the “Bio::Tools::Run::Phylo::PAML::Yn00” module in the BioPerl package.

### Identification of WGD, local and dispersed duplicates

In each investigated eudicot genome, the population of potential gene duplications was identified using BLASTP [42] with the following parameter: top five non-self matches and *E*<10^−10^. MCScanX [43] was applied to each BLASTP output. WGD duplicate pairs were the pairs of colinear genes generated by MCScanX. Potential local duplicate pairs were obtained from the “.tandem” files generated by MCScanX (within 10 annotated genes of each other). To reduce redundancy in potential local duplicate pairs, for each local duplicate, only the local duplicate pair with the smallest Ks was kept for analysis. Potential dispersed duplicate pairs were derived by excluding WGD duplicate pairs and potential local duplicate pairs from the population of potential gene duplications. To reduce redundancy in potential dispersed duplicate pairs, for each dispersed duplicate, only the dispersed duplicate pairs with smallest Ks was kept for analysis.

Specially in *Arabidopsis thaliana*, WGD duplicate pairs created by α, β and γ events were initially obtained from a previous study [3]. Then, α WGD duplicates were updated according to another study [44], to exclude tandemly-duplicated WGD duplicates which were shown to have very similar evolutionary patterns with local duplicates [45]. Tandem and proximal duplicate pairs were obtained from another previous study [33]. Potential dispersed duplicate pairs were then derived by excluding WGD duplicate pairs, tandem and proximal duplicate pairs from the population of potential gene duplications (generated by BLASTP as previously noted). To reduce redundancy in potential dispersed duplicate pairs, for each dispersed duplicate, only the dispersed duplicate pairs with smallest Ks was kept for analysis.

### Estimation of relocated γ duplicates in *Carica* and *Theobroma*

Phylogeny between *Carica* (or *Theobroma*) and outgroups is shown in Fig 2A. Gamma duplicates in *Carica* (or *Theobroma*) were initially the intra-genome colinear genes generated by MCScanX since no lineage-specific WGDs have occurred. We then excluded the duplicates without any colineary homologs in outgroups from the γ duplicates, i.e. to exclude recent segmental duplicates. Gene colinearity conservation between *Carica* (or *Theobroma*) and outgroups was used to estimate the epochs of *Carica* (or *Theobroma*) dispersed duplicates. Relocated γ duplicates in *Carica* (or *Theobroma*) were the dispersed duplicates which show colinearity with *Populus*, *Vitis* and/or *Solanum*, but do not show colinearity with monocots or *Musa*. We got 996 γ duplicates and 4024 relocated γ duplicates in *Carica*, and 4267 γ duplicates and 3906 relocated γ duplicates in *Theobroma*.

### Enrichment analysis

Enrichment analysis was performed by Fisher’s exact test.

### Gene function analysis

Gene functions in *Arabidopsis thaliana* were denoted by Gene Ontology (GO) terms, available from TAIR [46]. We selected the 29 GO terms belonging to “biological process” and with >300 *Arabidopsis thaliana* genes for further analysis. For each origin of duplicates, the fold enrichment for each selected GO term was the fraction of the duplicates of this origin having the GO term divided by the faction of the pooled duplicates having the GO term. Then, for each origin of duplicates, its functional profile was denoted by a vector consisting of 29 ratios, which correspond to the fold enrichment for the 29 selected GO terms. Functional similarity between different origins of duplicates was denoted by the Pearson’s correlation coefficient (*r*) of their functional profiles. To make the heat map of the functional relationships among different origins of duplicates, average linkage hierarchical clustering with distance = 1- *r* was employed.

### Phenotypic data

The phenotypic effects of 5,360 *Arabidopsis* mutant genes (by single locus knockout) were obtained from a published study [34], of which 1,742 showed phenotypic changes, whereas 3,618 did not.

### Protein-protein interaction data

We downloaded a dataset of genome-wide protein-protein interactions (*Arabidopsis* Interactome version 1 “main screen”, i.e. AI1-main) from a previous study, involving 5,664 binary interactions between 2661 proteins [36]. Note that AI1-main was derived from a population of 8,595 *Arabidopsis* genes. So when we compared the proportions of genes involved in PPIs among different gene groups, the genome background consisted of only these 8,595 genes.

### Gene expression and regulation analysis

Processed microarray data measured by the Affymetrix *Arabidopsis* ATH1 Genome Array (GPL198) were obtained from previous studies [12, 47]. The expression divergence between two genes was measured by 1-*r*, where *r* is the Pearson’s correlation coefficient between their expression profiles.

The promoter region of each *Arabidopsis thaliana* gene was defined as the DNA sequence between 600bp upstream and 200bp downstream of its transcription start site. A total of 155 position weight matrices (PWMs) for plant genomes were retrieved from the TRANSFAC [48] and JASPAR [49] databases. Based on these PWMs, FIMO [50], a DNA motif search tool, was executed to detect the matched PWMs for the promoter regions of each *Arabidopsis thaliana* gene. The gene regulation similarity between duplicated genes was defined as the fraction of their shared PWMs in their promoter regions. The regulation divergence between duplicated genes is computed by:

$$\text{Gene regulation divergence}=1-\frac{\left\{\text{PWMs in duplicate}\;1\}\cap \{\text{PWMs in duplicate}\;2\right\}}{\left\{\text{PWMs in duplicate}\;1\}\cup \{\text{PWMs in duplicate}\;2\right\}}.$$

## Supporting Information

S1 Fig Retention rates of *Arabidopsis lyrata* dispersed duplicates in different epochs. (PNG)

S2 Fig Retention rates of *Medicago truncatula* dispersed duplicates in different epochs. (PNG)

S1 Table *Arabidopsis thaliana* duplicated genes and their evolutionary origins. (XLSX)

S2 Table Functional enrichment analysis of *Arabidopsis thaliana* duplicated genes of different evolutionary origins. (XLSX)
