# Supplementary figures and images for: Large-Scale Gene Relocations following an Ancient Genome Triplication Associated with the Diversification of Core Eudicots
Source: PLoS One. 2016 May 19;11(5):e0155637. doi: 10.1371/journal.pone.0155637 (PMC4873151; doi:10.1371/journal.pone.0155637)

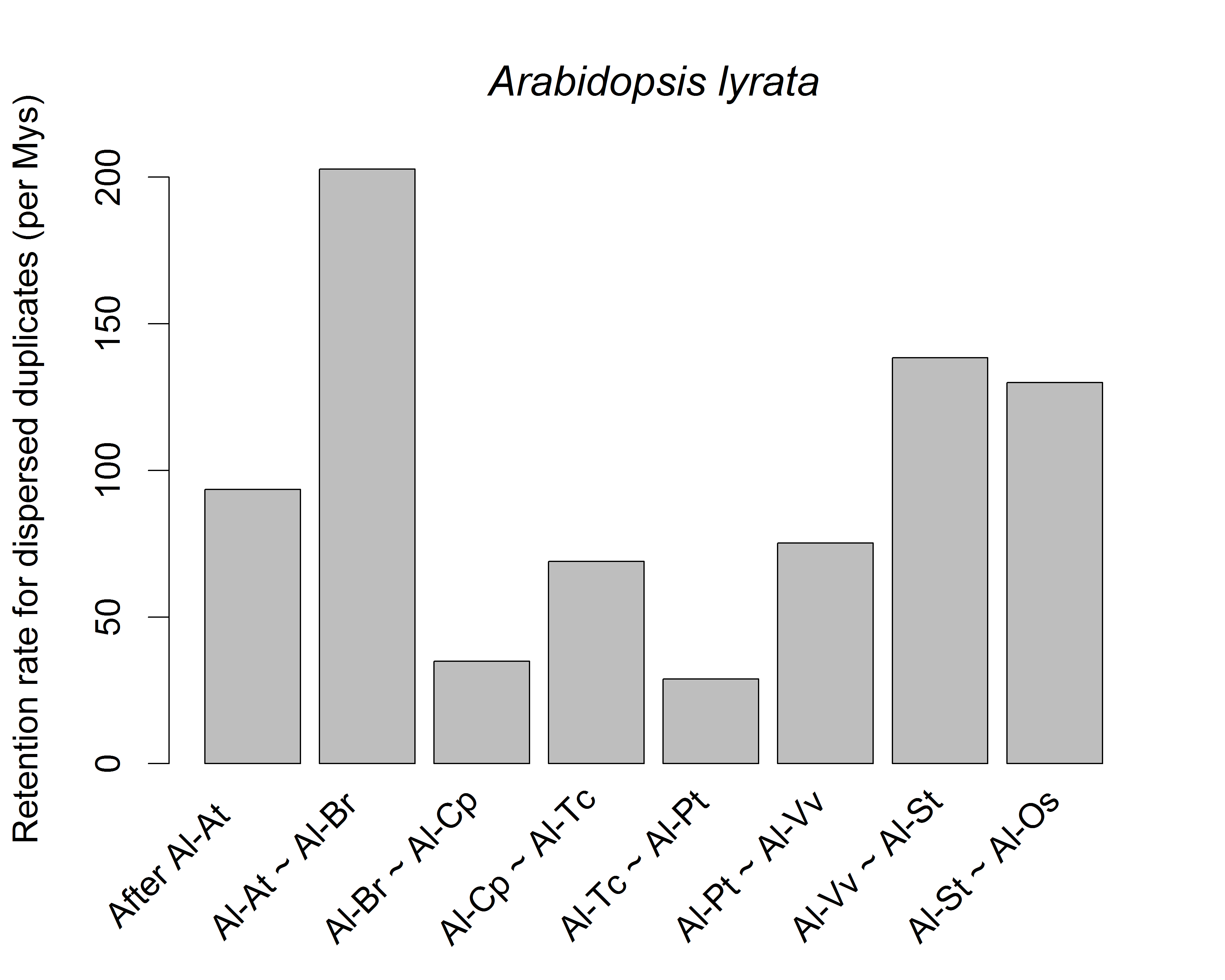

Supplement: S1 Fig — (PNG) [file pone.0155637.s001.png]

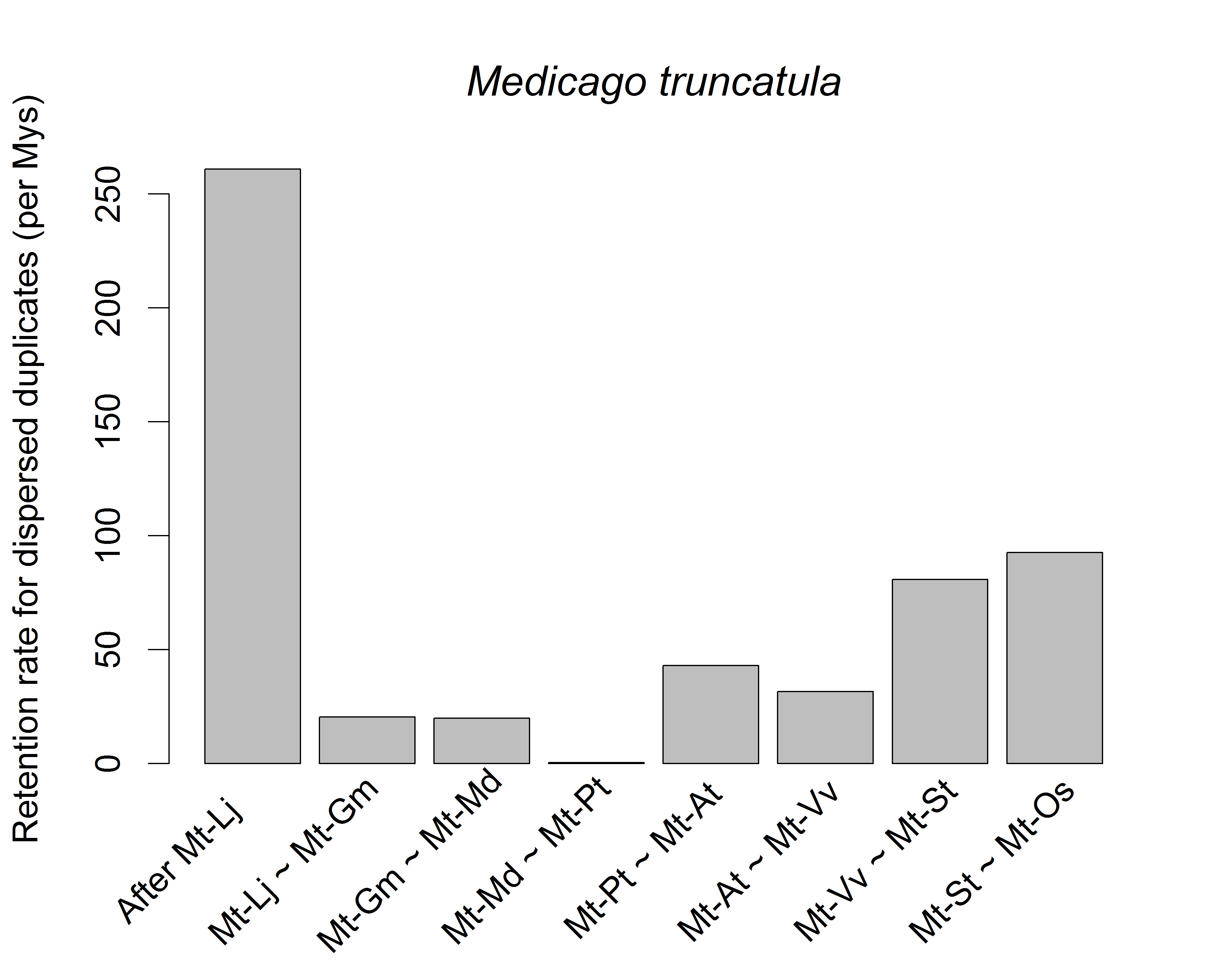

Supplement: S2 Fig — (PNG) [file pone.0155637.s002.png]
